# Theoretical Studies on the Quantum Capacitance of Two-Dimensional Electrode Materials for Supercapacitors

**DOI:** 10.3390/nano13131932

**Published:** 2023-06-25

**Authors:** Jianyan Lin, Yuan Yuan, Min Wang, Xinlin Yang, Guangmin Yang

**Affiliations:** College of Physics, Changchun Normal University, Changchun 130032, China

**Keywords:** electrical double-layer capacitors, 2D electrode materials, quantum capacitance, first-principle calculations

## Abstract

In recent years, supercapacitors have been widely used in the fields of energy, transportation, and industry. Among them, electrical double-layer capacitors (EDLCs) have attracted attention because of their dramatically high power density. With the rapid development of computational methods, theoretical studies on the physical and chemical properties of electrode materials have provided important support for the preparation of EDLCs with higher performance. Besides the widely studied double-layer capacitance (*C_D_*), quantum capacitance (*C_Q_*), which has long been ignored, is another important factor to improve the total capacitance (*C_T_*) of an electrode. In this paper, we survey the recent theoretical progress on the *C_Q_* of two-dimensional (2D) electrode materials in EDLCs and classify the electrode materials mainly into graphene-like 2D main group elements and compounds, transition metal carbides/nitrides (MXenes), and transition metal dichalcogenides (TMDs). In addition, we summarize the influence of different modification routes (including doping, metal-adsorption, vacancy, and surface functionalization) on the *C_Q_* characteristics in the voltage range of ±0.6 V. Finally, we discuss the current difficulties in the theoretical study of supercapacitor electrode materials and provide our outlook on the future development of EDLCs in the field of energy storage.

## 1. Introduction

In today’s world, non-renewable energy sources are decreasing. Energy supply is closely related to environmental issues and basic human needs. Improving the conversion efficiency of energy sources and developing new energy sources has become an urgent problem that needs to be solved in the midst of the energy crisis [1]. In this energy-dependent world, electrochemical devices for energy storage have played a crucial role in overcoming the depletion of fossil fuels [2]. Compared with conventional batteries, supercapacitors have the advantages of high power density, long cycle life, and fast charge and discharge rates. However, the energy density of supercapacitors is usually low, which is a major obstacle to their development [3]. Supercapacitors are usually classified into three types: (1) electrical double-layer capacitors (EDLCs) with ion adsorption through the electrode surface; (2) pseudocapacitors with surface Faraday redox reactions on the electrodes; (3) hybrid supercapacitors that are a mixture of the above two [4,5]. In this paper, we mainly focus on the electrode materials for EDLCs.

For the charging process on EDLCs, the anions and cations in the electrolyte are adsorbed to the positive and negative surfaces, respectively, forming a double-layer due to the external voltage difference. After charging, the anions on the double-layer produce a potential difference between the two plates to store energy. Discharging is the opposite process of charging. Because of the fast rate of this simple physical adsorption process, the EDLCs usually have high power densities. For the electrode materials, carbon materials with a large specific surface area and good electrical conductivity are generally the best choice for the fabrication of EDLCs [4,6,7,8].

EDLCs have high output power, fast charge and discharge rates, and long service lives but poor energy density [9,10,11]. Therefore, increasing the energy density of EDLCs has become a key research goal. The energy density of EDLCs is determined by the operating voltage and the specific capacitance of the electrode/electrolyte system [12]. The total interface capacitance (*C_T_*) of EDLCs is related to the quantum capacitance (*C_Q_*) and the double-layer capacitance (*C_D_*), with the expression of 1/*C_T_* = 1/*C_Q_* + 1/*C_D_* [13,14,15,16,17]. *C_Q_*, also known as electrode capacitance, reflects the finite quantum state process of the electron-filled system [18,19]. The theoretical prediction of increasing the total EDLC capacitance by increasing the *C_Q_* of the electrode material has been experimentally confirmed [20]. *C_Q_* is proportional to the density of electronic states. A large number of quantum states near the Fermi level can lead to a high *C_Q_*. The electronic structure of a material can be modified by changing the dopants, functional groups, defects, etc. of the structure, thus changing the specific surface area and surface morphology of the electrode material. The larger the specific surface area, the better the energy storage performance of the electrode material [21,22].

Usually, materials with a thickness of a few atomic layers are considered as two-dimensional (2D) materials [3]. Since the discovery of graphene in 2004, 2D materials have experienced rapid development. For example, 2D carbon materials demonstrate excellent properties, such as high specific surface area and high electrical conductivity [23]. Since the working mechanism of EDLCs is an electrostatic effect, the anions and cations on the electrode material surface move to the positive and negative electrodes during the charging and discharging process, forming electric double-layers at the interface. Thus, 2D electrode materials with larger specific surface areas are more suitable for EDLCs than their 3D counterparts [24,25,26,27]. However, 2D electrode materials are highly susceptible to stacking due to their high edge activity, resulting in a decreased specific surface area and capacity during reagent application. Numerous studies have been devoted to maintaining or increasing the specific surface area of the electrode materials and improving the circulation rate of anions and cations by changing the morphology of the electrode surface. In this paper, we focus on the aforementioned modification measures (defects, doping changes, adsorption of functional groups, etc.) and atomic exchange on the electrode materials. We summarize the classification of electrode materials and highlight the materials with better performance and greater potential for experimental application.

## 2. Theoretical Basis

Among many low-dimensional materials, the differential quantum capacitance (*C_diff_*) can be defined by

(1) $$C_{diff}=\frac{d\sigma }{d\Phi_{G}}=e^{2}DOS\left(-V_{e}\right)$$

in which *dσ* and *dΦ_G_* represent the differential charge density and differential local potential, respectively.

Thus the magnitude of *C_diff_* is dependent on the density of states. The density of states is essentially the number of different states that an electron is allowed to occupy at a given energy level, i.e., the number of electron states per unit volume of energy. Due to quantum confinement effects and the limitation of the low density of states, the significant movement of Fermi levels in two-dimensional materials could accumulate a sufficient number of carriers to provide better energy density, thus improving the performance of supercapacitors.

The excess charge density can be expressed as:

(2) $$∆Q=\int_{-\infty }^{+\infty }D\left(E\right)\left[f\left(E\right)-f\left(E-e\phi_{G}\right)\right]dE$$

where *D*(*E*) represents the density of states of the system, ƒ(*E*) is the Fermi-Dirac distribution function, *E* is the electronic energy with respect to the Fermi level, and *e* is the fundamental charge. For 2D materials, *C_diff_* can be obtained by the following equation:

(3) $$C_{diff}=e^{2}\int_{-\infty }^{+\infty }D\left(E\right)F_{T}\left(E-e\phi_{G}\right)dE$$

where *F_T_*(*E*) is the thermal spreading function, which is obtained from the following:

(4) $$F_{T}\left(E\right)={\left(4\kappa_{Β}Τ\right)}^{-1}{sech}^{2}\left(\frac{E}{2\kappa_{Β}Τ}\right)$$

It is also common for researchers to analyze the energy storage capacity of supercapacitors by calculating *C_int_*. The *C_int_* is obtained by integrating the *C_diff_* over the charge and discharge cycles [28,29].

(5) $$C_{int}(V)=\frac{Q}{V}=\frac{1}{V_{e}}\int_{0}^{V}C_{diff}(V^{\prime })dV^{’}$$

In this paper, *C_diff_* is equally defined as *C_Q_*.

## 3. Research Progress of 2D Electrode Materials for EDLCs

### 3.1. Graphene-like 2D Main Group Elements and Compounds

Graphene with sp^2^ hybridization is a typical representative of 2D materials. [30] In the past decade, graphene-based electrode materials have become a popular research direction for supercapacitor electrode materials. More recently, scientists have tried to discover various analogs with six-membered ring structures that are similar to graphene, such as silicene, germanene, phosphorene, etc. On the other hand, the 2D main group compounds with graphene-like structures, such as 2D carbon nitride (CN), have also been synthesized in recent years. There are also many studies on the performance of 2D CNs as electrode materials.

#### 3.1.1. Graphene

Graphene is prone to stacking due to its high edge activity, resulting in a decrease in specific surface area and capacity. Since graphene oxides are usually used as precursors for graphene preparation, there are many vacancies in graphene-based materials. Many studies have focused on maintaining the dispersion of graphene [31,32].

**Figure 1:**
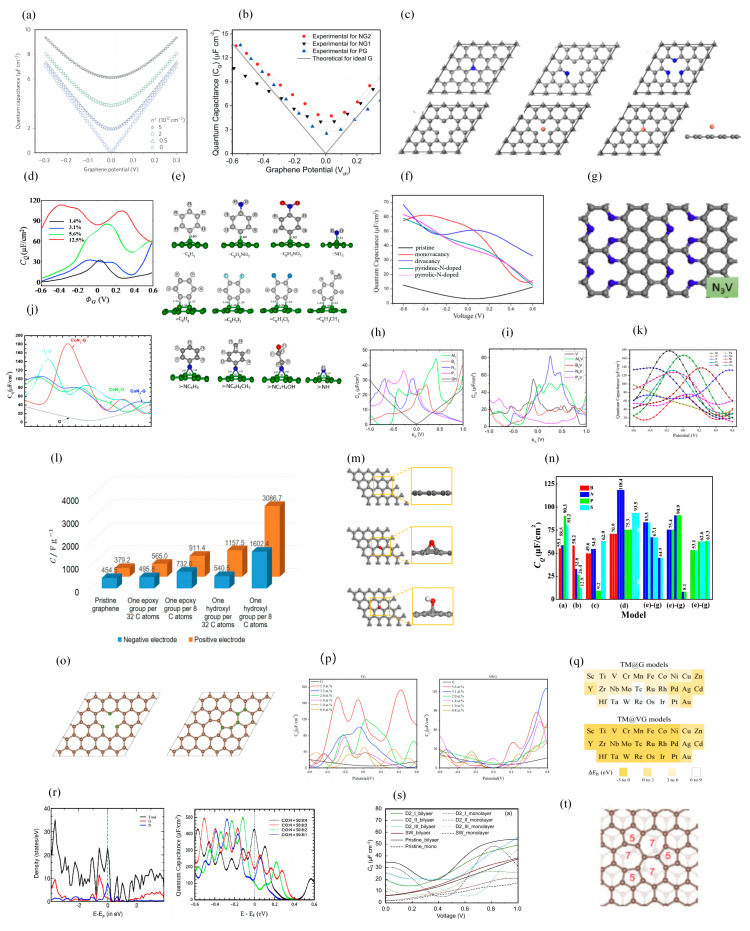
(**a**) *C_Q_* versus potential curves for single- and double-layer graphene. (**b**) Dependence of *C_Q_* on N-dopant concentration as a function of graphene potential (V_ch_). (**c**) The structures of six defective graphene models. (**d**) *C_Q_* maps of the graphene model with different N-doping concentrations. (**e**) Schematic structures of monovalent, divalent, and trivalent functional groups. (**f**) *C_Q_-V* curves of pristine, cavity, pyridinic-N-doped and pyrrolic-N-doped graphene. (**g**) Structure of 3N-doped and single-vacant graphene (N_3_V). (**h**,**i**) *C_Q_* versus potential plot for X_1_ and X_3_V monolithic graphene structures. (**j**) *C_Q_* versus potential plot for CoN*_x_* (*x* = 1, 2, 3, 4) co-doped graphene. (**k**) *C_Q_* versus potential plot for graphene doping within the transition metal plane. (**l**) The maximum *C_Q_* in the potential range of −1.5 V~1.5 V for graphene with different oxygen-containing group concentrations at the negative and positive electrodes. (**m**) Optimized structures of pristine, epoxy graphene and graphene oxide containing hydroxyl groups. (**n**) Change trend chart of the maximum value of *C_Q_* for the B(N, P, S)-doped graphene with different doping models (model-a, model-b, model-c, and model-d) and the N/S, N/P-co-doped the supercell 4 × 4 graphene with different models (model-e, model-f, and model-g). (**o**) Structures of vacancy-defected (VG) and Stone–Wales defected graphenes (SWG). (**p**) *C_Q_* of VG and SWG at different concentrations. (**q**) The stability of TM@G and TM@VG models. (**r**) PDOS and *C_Q_* versus potential curves for N, O co-doped graphene. (**s**) *C_Q_* versus potential for monolayered and bilayered graphene with different defects. (**t**) Configuration of defective bilayer graphene containing D2_11 type (555–777) point defects.

In 2009, Xia et al. measured the *C_Q_* of monolayer and bilayer graphene, and the curve of *C_Q_*-potential was symmetrical and v-shaped (Figure 1a) [33]. Many scientists have experimentally demonstrated that the capacitance of carbon electrodes can be improved by doping nitrogen (N) atoms or the functionalization of N-containing groups [34,35,36,37,38,39,40,41]. Zhang et al. demonstrated that N-doping changes the electronic structure of graphene and increases the carrier density, which changes the *C_Q_* and leads to an increase in the interfacial capacitance value (Figure 1b) [42]. Yang et al. theoretically investigated the effects of N-doping configuration, N-doping concentration, vacant concentration, and transition metal atoms (Cu, Ag, Au) adsorption on the electronic structure and *C_Q_* of graphene [14]. Their results show that N-doping, vacancy defects, and transition metal atom adsorption can significantly enhance the *C_Q_* of graphene. Among them, the maximum value of *C_Q_* increases from 32.68 to 113.1 μF/cm^2^ as the N-doping concentration increases from 1.4% to 12.5% (Figure 1c,d). Mousavi-Khoshdel et al. investigated the changes in *C_Q_* of functionalized graphene with monovalent functional groups (-C_6_H_5_, -C_6_H_4_NH_2_, -C_6_H_4_NO_2_, -NH_2_) and divalent functional groups (-C_6_H_4_, -C_6_H_2_F_2_, -C_6_H_2_Cl_2_, -C_6_H_3_CH_3_) [43]. Their results show that the *C_Q_* values of functionalized graphene are higher than that of pristine graphene in both cases. A schematic diagram of the structures of the three types of groups is shown in Figure 1e. Chen et al. investigated the interaction and *C_Q_* of N and S co-doped graphene [44]. The maximum *C_Q_* of pristine graphene is 14 μF/cm^2^ and the minimum *C_Q_* is 2.5 μF/cm^2^. The *C_Q_* values of pyridine-N-doped graphene and pyrrolic-N-doped graphene at the Fermi level are about 41.4 and 38.2 μF/cm^2^, respectively. In a subsequent study, they found that the *C_Q_* value of N/S co-doped graphene could be higher than that of single N-doped graphene, with the highest *C_Q_* value being 95.8 μF/cm^2^. However, the *C_Q_* does not improve more when another N or S atom is added to the co-doped system (Figure 1f).

Hirunsit et al. studied the *C_Q_* variation of Al-, B-, N-, and P-doped single-vacancy (V) and multilayer graphene. [28] They showed that Al_1_, V, Al_3_V, and N_3_V modification can increase the *C_Q_* by a large amount (>40 mF/cm^2^), and the N_3_V structure showed the highest *C_Q_* value, which was 82.18 mF/cm^2^ (0.26 V). The construction of multilayer graphene also improves the *C_Q_* (Figure 1g–i). Hu et al. investigated the effect of transition metal (Mn, Fe, Co, Ni) and N atom (TMN*_x_*, *x* = 1–4) co-doping on the *C_Q_* of graphene [45]. The co-doped systems showed an increase in *C_Q_*, with a maximum value of 180.50 μF/cm^2^ for CoN_2_ g at −0.3 V (Figure 1j). A similar study was carried out by Wang et al., who explored the *C_Q_* changes of transition metals after in-plane doping and out-of-plane doping on graphene [46]. Their conclusions show that the *C_Q_* of in-plane doping is larger than that of out-of-plane doping, where the charge (*Q*) of Sc-doped graphene could reach 85 μC/cm^2^ at negative bias (Figure 1k). Song et al. studied the variation of *C_Q_* of epoxy (- O -)- and hydroxy (-OH)-modified graphene oxide [47]. The results show that the modified graphene oxide also has a higher *C_Q_* than the original structure. There is a significant increase of *C_Q_* with the increasing oxidation degree on both positive and negative bias (Figure 1l,m).

Sruthi et al. found that the *C_Q_* of graphene can be significantly enhanced by doping on the pristine graphene surface with N, Cl, and P atoms [48]. Additionally, very large *C_Q_* (>600 μF/cm^2^) can be achieved when doping N, Cl, and P atoms near room temperature. Xu et al. investigated the *C_Q_* of graphene doped/co-doped with B, N, P, S atoms and vacancy [49]. They also obtained the *C_D_* in a classical 1 M NaCl aqueous solution by using molecular dynamics simulations. Then, the *C_T_* was calculated. Graphene that has been 3N-doped with a single vacancy is supposedly the best candidate as an EDLC electrode (Figure 1n). Zhou et al. investigated the effects of doping (B, N, Al, Si, P, S), vacancies, and Stone–Wales defects on the *C_Q_* of graphene and found that Stone–Wales defects could also improve the *C_Q_* of graphene, but not better than doping or vacancy. The maximum *C_Q_* of Si-VG is 169.76 μF/cm^2^ at −0.29 V. The maximum *C_Q_* is 168.90 μF/cm^2^ at −0.06 V, when the VG concentration is 5.9% (Figure 1o,p). Zhang et al. determined a variety of materials suitable for supercapacitor applications by systematic calculations and generalizations [50]. They explored the *C_Q_* of 56 species of transition metal atoms and vacancy-doped/co-doped graphene, named TM@G and TM@VG, respectively (Figure 1q). Sruthi et al. explored the effect of different co-doping ratios on the *C_Q_* of graphene [51]. When the dopant ratio C:O:N is 50:8:4, the *C_Q_* of the system at the Fermi energy level can reach 423.73 μF/cm^2^ (Figure 1r).

One of the inevitable problems in manufacturing and using graphene materials is the stacking of layers, which significantly affects the structure the electrochemical properties. Cui et al. explored the effect of stacking on multilayered graphene [52]. They assumed a two-layer ab-stacked graphene model, where the top layer is defective and the bottom layer is perfect. They showed that the *C_Q_* of the pristine bilayer graphene increases linearly with voltage, reaching a maximum value of 37.7 μF/cm^2^ at 1.0 V. The peak of D2_III has a maximum *C_Q_* of 56.1 μF/cm^2^ at a voltage of 1 V (Figure 1s,t). Zhou et al. explored co-doping with N, P, S and transition metals (Ti, V, Cr, Mn, Co, Ni) in monolayer and multilayer graphene [53]. Their study showed that doping with transition metals (TM) improves the *C_Q_* more than co-doping with N, P, and S, and the Ti/Ni and N/P/S co-doped systems exhibit excellent *C_Q_*. However, the *C_Q_* of the multilayer system decreases due to the interactions between the adjacent layers of dopants. In a study by Zeng et al., it was found that the capacitance of B (N)-doped graphene as an anode (cathode) can reach a record *C_Q_* of 4317 F/g (6150 F/g) [54].

#### 3.1.2. Silicene

Inspired by graphene, silicene is made from 2D layered nanosheets. Silicene sheets with different structures have been successfully synthesized on various substrates. Silicene with a buckling layer structure has a high surface area [55]. It is considered as an excellent anode material for Li-ion batteries because it has enough space to adsorb Li-ions and prevents structural breakage induced by the insertion of Li-ions. Similar to graphene, it is also expected to be one of the ideal electrodes for EDLCs.

**Figure 2:**
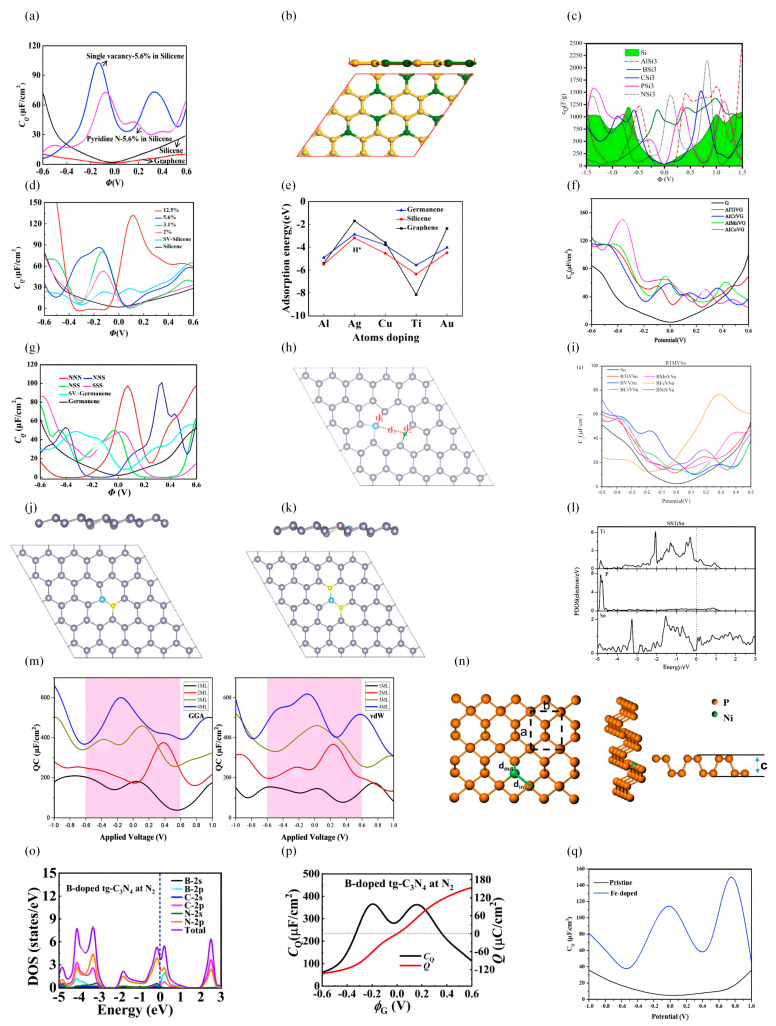
(**a**) *C_Q_* versus potential plot for single vacant and pyridine-N-doped silicene. (**b**) Schematic diagram of the atomic structure of 2D AlSi_3_. (**c**) *C_Q_* versus potential curves for pristine and X-doped silicene (X = Al, B, C, P, N). (**d**) *C_Q_* versus potential plot for single vacant silicene adsorbed with different Ti concentrations. (**e**) Adsorption energy of metal atoms on defective germanene, silicene, and graphene. (**f**) The plot of *C_Q_* versus potential for defective germanene co-doped with Ti, Cr, Mn, Co, and Al. (**g**) *C_Q_* versus potential curves for N/S co-doped single vacant germanene. (**h**) Structure of B and transition metal atoms co-doped with defective stanene. (**i**) *C_Q_* versus potential plot for BTMVSn. (**j**,**k**) Structures of S and Ti atoms co-doped and line-doped stanene. (**l**) TDOS diagram of SSTiSn. (**m**) *C_Q_* diagrams of multilayered boronene. (**n**) Structure of Ni-doped phosphorene. (**o**,**p**) DOS and *C_Q_* diagrams of B-doped *tg*-C_3_N_4_. (**q**) *C_Q_* versus potential plots for iron-doped BC_3_ monolayer.

Yang et al. explored the effects of vacancy and dopants (N, P, B, and S) concentration on the *C_Q_* of silicene [56]. Their results show that the maximum *C_Q_* of silicene increases with the defect concentration from 1.91 μF/cm^2^ at −0.38 V to 102.65 μF/cm^2^ at −0.19 V. When the pyridine-N doping concentration is 5.6%, the maximum *C_Q_* is 73.28 μF/cm^2^ (−0.07 V). The *C_Q_* is higher than that of the pristine silicene in all modified structures (Figure 2a). Momeni et al. explored the *C_Q_* of pristine silicene, defective silicene, and XSi_3_-like silicene (X = Al, B, C, N, P) structures [57]. Their results show that the alternative doped XSi_3_-like silicene structures have higher *C_Q_* compared to pristine silicene (*C_Q_* = 1200 F/g) and graphene (*C_Q_* = 500 F/g). The AlSi_3_ system reaches a maximum *C_Q_* of 2573 F/g under positive bias. They also showed that the large *C_Q_* of XSi_3_-like silicene originates from the high electronic states at the Fermi level of 2p and/or 3p orbitals of X and Si atoms, as evidenced by projected density of state analysis (Figure 2b,c). Xu et al. explored the *C_Q_* of silicene with metal atom (Ti, Au, Ag, Cu, and Al atoms) adsorption and single-vacancy doping. [58] It was found that a single vacancy with metal adsorption can significantly increase *C_Q_*. When the Ti concentration is increased from 2% to 12.5%, the maximum value of *C_Q_* increases from 52.2 μF/cm^2^ at −0.12 V to 132.2 μF/cm^2^ at 0.12 V (Figure 2d).

#### 3.1.3. Germanene

Silicene and Germanene are of great interest as 2D layered nanosheet materials inspired by graphene. Germanene is more prominent than silicene and graphene in terms of its spin–orbit interaction. The large spin–orbit gap (24 meV) of germanene makes it a typical alternative material with the quantum spin Hall effect [59,60,61,62,63]. Moreover, germanene is more easily to be functionalized and has been synthesized by different chemical methods [64,65,66,67]. In order to further investigate the electrochemical properties of germanene and probe for more superior performance electrode materials, numerous researchers have investigated the *C_Q_* of germanene with doping, co-doping, and vacancy defects.

Si et al. explored the effect of single vacancy (SV), adsorption of Ti, Au, Ag, Cu, Al atoms, and different doping concentrations on the *C_Q_* of germanene [61]. Similar to graphene and silicene, vacancies can increase the *C_Q_* of germanene, especially in the positive bias range. The *C_Q_* of Ti- and Cu-doped SV germanene is superior to that of Au-, Ag-, and Al-doped ones. Moreover, Ti-doping is more stable in graphene, silicene, and germanene (Figure 2e). Zhou et al. found that transition metal (Ti, Cr, Mn, and Co) doping enhanced the *C_Q_* better than B/N/Al doping [68]. The maximum *C_Q_* can reach 91.47 μF/cm^2^ (0.2 V) for Ti-doping near the Fermi level. The co-doped system improves *C_Q_* more than single-doping (Figure 2f). Si et al. further investigated the effects of doping/co-doping, vacant defects, and multilayer structure on the electronic structure and *C_Q_* of germanene [69]. Their results show that N-doping can significantly improve the *C_Q_* of germanene. In a study of single and multilayered germanene co-doped with NAl, NNAl, NPAl, and NSAl, it was found that the interlayered interactions contributed more to the increase in *C_Q_* (Figure 2g).

#### 3.1.4. Stanene

Stanene is a novel material that has received increasing attention in recent years. It has been successfully realized by epitaxial growth on Bi_2_Te_3_ (111) substrates [70,71,72]. Stanene exhibits several remarkable features, including large spin–orbit gaps, topological superconductivity, quantum anomalous Hall behavior, giant magnetoresistance, and efficient thermoelectricity [73]. Additionally, there have been numerous studies showing that measures such as doping with metal atoms can have a large effect on the structural and electrochemical properties of Stanene [74,75].

Zhou et al. verified the effect of vacancies and the single-doping and co-doping of light element atoms (B, N, Al, Si, P, S) and transition metals (Ti, V, Cr, Mn, Fe, Ni) on the geometry, electronic structure, and *C_Q_* of stanine [76]. Their results show that vacancy, doping, and co-doping can improve the *C_Q_* of stanene and that co-doped defective stanene exhibits better *C_Q_* at negative potentials than at positive bias, indicating that it can be used as a good anode material. The maximum *C_Q_* of BFeVSn is 76.52 μF/cm^2^ (0.29 V) under positive bias conditions (Figure 2h,i). Zhou et al. also investigated the effect of N/P/S and line co-doping with heavy metals (Ti, V, Fe, Ni) on stanine [77]. The effect of line co-doping to improve the *C_Q_* of the system is more obvious, where the maximum *C_Q_* at a positive bias of SSTiSn is 77.18 μF/cm^2^, which could be attributed to the increased electronic states of the Ti dopant and adjacent Sn atoms (Figure 2j–l).

#### 3.1.5. Boronene

Due to the high carrier concentration, boronene has been used in plasma devices, extending the functionality to the visible region. Boronene is predicted to be an excellent candidate for Li-ion batteries due to its high Li capacity [78,79,80].

Kolavada et al. theoretically analyzed the *C_Q_* of δ-6 boronene with different layer numbers in aqueous electrolytes (AEs) and ionic liquid electrolytes (ILEs) [81]. In both AE and ILE systems, *C_Q_* enhances as the number of layers increases from 1 monolayer (ML) to 4 ML. When the number of layers is 4 ML, *C_Q_* can reach more than 600 μF/cm^2^ in both systems (Figure 2m).

#### 3.1.6. Phosphorene

Phosphorene is a relatively new member to the group of 2D materials discussed in this study. Its strong in-plane anisotropy makes phosphorene a unique material for novel electronic devices [82,83,84,85,86]. Zu et al. fabricated supercapacitors by using phosphorene as electrodes and with the discharge capacity of 3181.5 F/g in a three-electrode configuration [87].

Ramesh et al. computationally examined the effect on phosphorene when half-metal (Si)-dopants, active-nonmetal (S)-dopants, and two transition metal (Ti, Ni) dopants replace the P atom [88]. The *C_Q_* of pristine phosphorene is approximately symmetric, with a minimum value of 2.27 μF/cm^2^ at the Fermi level. The *C_Q_* of all substitution systems is higher than that of the pristine phosphorene, with the highest *C_Q_* value of 92.1 μF/cm^2^ for Ti-doping at 0.4 V (Figure 2n).

#### 3.1.7. Main Group Compounds

In recent years, four 2D carbon nitride (CN) structures, *hg*-C_3_N_4_, *tg*-C_3_N_4_, C_2_N, and C_3_N have been experimentally synthesized to further enrich the 2D electrode materials for supercapacitors [89,90,91]. These CN structures have high specific surface areas and excellent electrochemical stability. Therefore, CNs are considered good electrode materials.

Chen et al. investigated the effects of B and O doping on the electronic properties and *C_Q_* of 2D CNs. They found that doping with B or O could convert CNs from semiconductors to metals, thus improving the electrical conductivity [92]. The *C_Q_* values of B-doped CNs are all higher than those of B-doped monolayer graphene. The increased *C_Q_* can mainly be attributed to the strong hybridization between the dopant and the adjacent C and N atoms (Figure 2o,p).

Majdi et al. investigated the electrochemical properties of a new 2D Fe-doped boron carbide monolayer (FBC_3_ML) [93]. The maximum *C_Q_* of FBC_3_ML increases to 150.09 μF/cm^2^ compared to the original BC_3_ML, and the *C_Q_*-*V* curve becomes symmetric (Figure 2q).

### 3.2. Transition Metal Carbides or Nitrides (MXenes)

MXenes are 2D-layered materials derived from transition metal carbides, nitrides, or carbonitrides [94]. MXenes can be produced by selectively removing the A-layer from MAX phases, the 3D precursors of MXenes, noted as M*_n_*_+1_AX*_n_* phases (*n* = 1, 2 and 3). MAX phases are generally divided into three types: 211, 312, and 413 structures. M denotes early transition metal elements (such as Sc, Ti, Zr, Hf, V, Nb, Ta, Cr, Mo, etc.); A denotes elements of group 13 or 14, such as Al or Si; and X refers to C, N, or their mixtures [2]. MXenes are often used in the field of energy storage because of their special physical and chemical properties.

The first MXene, Ti_3_C_2_, was isolated from Ti_3_AlC_2_ powder by immersing it in a hydrofluoric acid solution [94]. Subsequently, many MXene family members have been synthesized using selective etching methods and many new MXene structures have been theoretically predicted [2]. When performing chemical etching methods, the metal atoms on the MXene’s surface can easily react with -H, -O, -F, and -OH groups in solution and terminate them on the MXene’s surface, thus giving rise to functionalized MXenes, M*_n_*_+1_X*_n_*T*_x_*, where T is the surface termination group [95,96,97]. This surface functionalization usually has an impact on the energy storage capacity of MXenes. Since the MAX phases usually have carbon vacancies (V_C_) [98,99], MXenes derived from the MAX phases are considered to have the same nature of carbon vacancies. The treatment of MXene materials via doping and vacancy also cause changes in the *C_Q_* of the materials. The current research on the *C_Q_* of MXene materials after modulation is more comprehensive. In this section, the effect of various modulation means on the *C_Q_* performance of MXene electrode materials will be discussed separately according to the different M elements.

#### 3.2.1. Ti_n+1_C_n_T_x_

As the first successfully prepared MXene, Ti_3_C_2_ and its isomer Ti_2_C have received increasing attention as electrode materials for supercapacitors [100,101].

**Figure 3:**
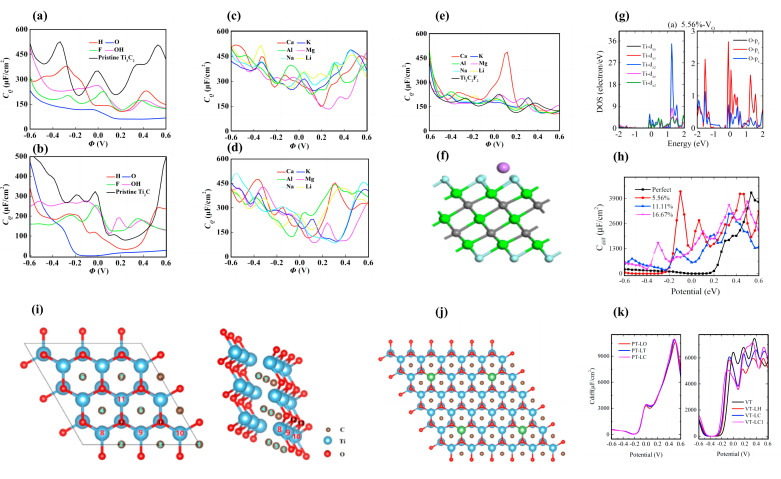
(**a**,**b**) *C_Q_* diagrams of functionalized Ti_3_C_2_ and Ti_2_C. (**c**,**d**) *C_Q_* diagrams of metal atoms-adsorbed Ti_3_C_2_ and Ti_2_C. (**e**) *C_Q_* diagram of metal atoms-adsorbed Ti_3_C_2_F_2_. (**f**) Structure of Ti_3_C_2_F_2_ adsorbed by metal atoms. (**g**) PDOS diagram of Ti_2_CO_2_ with an oxygen vacancy concentration of 5.56%. (**h**) *C_Q_* diagram of Ti_2_CO_2_ with different oxygen vacancy concentrations. (**i**) Schematic diagram of the vacant defected Ti_2_CO_2_ structure by removing atoms with atomic numbers (2), (3, 4), (1, 5, 6) and (3, 5, 7), denoted as CVL1, CVL2, CVL3, and CVL4, respectively. (**j**) The optimized structures of PT-LT monolayer. The red, light blue, brown, and light green balls denote the oxygen, titanium, carbon, and lithium atoms, respectively. (**k**) *C_Q_* diagrams of Li atoms adsorbed on Ti_2_CO_2_ (PT) and C-vacant Ti_2_CO_2_ (VT) monolayers.

Si et al. focused on the modulation of the two Ti-C MXenes materials using doping, vacancy, and adsorption methods [102]. Their calculations show the pristine structures have higher *C_Q_* compared to the functionalized Ti_3_C_2_ and Ti_2_C. The *C_Q_* of the functionalized structures decrease in an order of OH > F > H > O. The maximum *C_Q_* of OH groups adsorbed on Ti_3_C_2_ and Ti_2_C near the Fermi level is 264.414 μF/cm^2^ (0 V) and 276.960 μF/cm^2^ (−0.12 V) (Figure 3a,b). On the other hand, the adsorption of metal atoms on the surface of Ti_3_C_2_ and Ti_2_C can also change their *C_Q_* considerably. It is shown that on Ti_3_C_2_, the adsorption of Al atoms significantly increases the *C_Q_* (398.193 μF/cm^2^ at −0.072 V) due to the increase of the low potential local electronic states. A similar trend also appears for Ti_2_C, where Ti_2_C-Al has a maximum *C_Q_* of 444.192 μF/cm^2^ (0.312 V) near the Fermi level (Figure 3c,d). Furthermore, they have found that the adsorption of Ca atoms on Ti_3_C_2_F_2_ significantly improves the energy storage performance of the system with a *C_Q_* value of 488.153 μF/cm^2^. Nevertheless, the *C_Q_* of the Ti_2_C system shows no clear change (Figure 3e,f) [102].

Bafekry et al. investigated the oxygen vacancies in the Ti_2_CO_2_ monolayer and confirmed its semi-metallic properties by calculating the density of states of the system [103]. To further investigate the effect of O vacancy concentration on the properties of Ti_2_CO_2_, Su et al. systematically explored the *C_Q_* of Ti_2_CO_2_ with different O vacancy concentrations [104]. Their results demonstrate that the O vacancy concentration has a strong effect on *C_Q_*. The pristine Ti_2_CO_2_ has a low *C_Q_* under negative bias, with a maximum value of 4204.4 μF/cm^2^ (0.53 V). When one oxygen vacancy (5.56%) is introduced, the maximum *C_Q_* values increase to 4263.85 μF/cm^2^ (−0.1 V) and 4142.0 μF/cm^2^ (0.43 V) for negative and positive potentials, respectively. However, the maximum *C_Q_* decreases when two or three oxygen vacancies are introduced. The DOS of Ti_2_CO_2_ near the Fermi level mainly originates from O-p_z_ and Ti-d orbitals. They speculate that the introduction of oxygen vacancies increases the charge transfer between adjacent O and Ti atoms (Figure 3g,h) [104]. While Li et al. theoretically investigated the *C_Q_* of Ti_2_CO_2_ monolayer with carbon vacancy line (CVL) [105]. The introduction of CVL improved the *C_Q_* and *Q* of the system under negative and positive bias within ±0.6 V. The CVL4 system improves the maximum *C_Q_* of 469.7 μF/cm^2^ under negative bias compared to the pristine Ti_2_CO_2_ monolayer (Figure 3i). Li et al. calculated the *C_Q_* of pristine Ti_2_CO_2_ (PT) and C-vacant Ti_2_CO_2_ (VT) monolayers adsorbed by Li atoms. [106] From their results, it can be seen that the maximum *C_Q_* of PT-LT monolayer is 10,993 μF/cm^2^ (0.48 V), the maximum *C_Q_* of VT monolayer is 7592 μF/cm^2^ (0.36 V), and the maximum *C_Q_* of VT-LC1 is 6866 μF/cm^2^ (0.54 V), within the positive bias voltage (Figure 3j,k).

#### 3.2.2. Sc_n+1_C_n_T_x_

Sc-based MXenes have the lightest M atom. Among them, Sc_2_CF_2_ is a semiconductor with strong anisotropic carrier mobility and thermal conductivity. The electron mobility of Sc_2_CF_2_ in the zigzag direction is almost four times higher than that of phosphorene in the armchair direction, and its thermal conductivity is higher than that of most low-dimensional metals and semiconductor materials. Due to its excellent properties in electronic devices, Sc_2_CF_2_ has received much attention in recent years [107,108].

**Figure 4:**
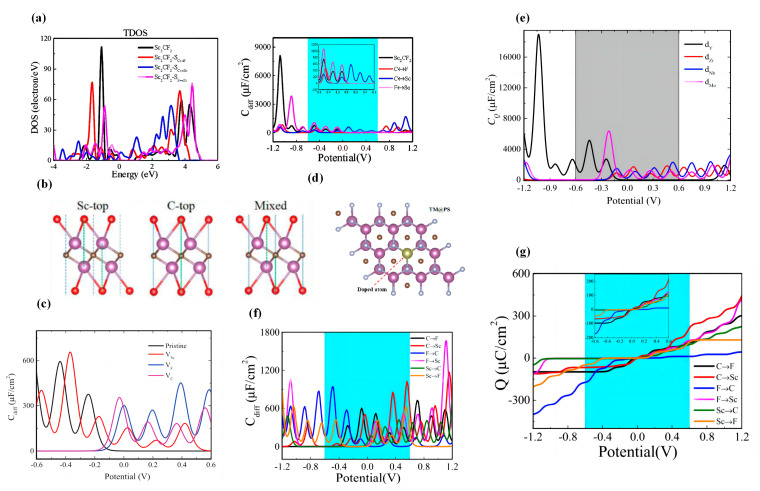
(**a**) TDOS and *C_Q_* of Sc_2_CF_2_-SC↔F, Sc_2_CF_2_-SC↔Sc, and Sc_2_CF_2_-SF↔Sc atomic exchange monolayers. (**b**) Three possible configurations of Sc_2_CT_2_, namely Sc-top, C-top, and hybrid configurations. (**c**) *C_Q_* of three vacancy-defective systems formed by removing a C, F, or Sc atom from Sc_2_CF_2_. (**d**) Schematic diagram of Sc_2_CF_2_ replacing one Sc atom for doping. (**e**) *C_Q_* versus potential plot for Sc_2_CF_2_ doped with 4d TM atoms (including Y, Zr, Nb, and Mo). (**f**) The plot of *C_Q_* versus potential for atomic exchanged Sc_2_CF_2_. (**g**) Charge versus potential for atomic exchanged Sc_2_CF_2_.

Cui et al. studied the exchange defects of Sc/F, Sc/C, and C/F atoms (Figure 4a) [109]. Their study shows that the atomic exchange has little effect on the semiconducting properties. Additionally, the maximum *C_Q_* of pristine Sc_2_CF_2_ under negative bias is 739.39 μF/cm^2^ (−0.48 V). The atomic exchange of C/F atoms and C/Sc atoms reduces the maximum *C_Q_* of Sc_2_CF_2_ monolayer under negative bias to 488.60 μF/cm^2^ (−0.44 V) and 282.57 μF/cm2 (−0.48 V), respectively. The atomic exchange between F and Sc atoms increases the maximum *C_Q_* of Sc_2_CF_2_ monolayer under negative bias to 1037.76 μF/cm^2^ (−0.48 V). Cui et al. also studied the *C_Q_* of Sc_2_CT_2_ (T = F, P, Cl, Se, Br, O, Si, S, OH) monolayers (Figure 4b) [110]. They found that the maximum *C_Q_* of the Sc_2_C monolayer in aqueous electrolyte (±0.6 V) is 1025.01 μF/cm^2^ and 1297.03 μF/cm^2^ under negative and positive bias, respectively. Compared with the original Sc_2_C structure, the maximum *C_Q_* under the negative bias of Sc_2_CT_2_ (T=P, Cl, Se, Si) increases, with the maximum *C_Q_* of Sc_2_CP_2_ being 3800.34 μF/cm^2^. The maximum *C_Q_* of the Sc_2_CSi_2_ monolayer increases to 1708.82 μF/cm^2^ under positive bias voltage, but the maximum *C_Q_* of all other systems decreases under the positive bias voltage. However, all of them have a larger maximum *C_Q_* than the system with Sc_2_CF_2_ for atomic exchange.

Subsequently, Cui et al. theoretically studied the *C_Q_* of Sc_2_CF_2_ with different atomic vacancies (Figure 4c) [111]. They observe that, at positive bias, pristine Sc_2_CF_2_ has almost no *C_Q_*, while the introduction of vacancies increases it. The maximum *C_Q_* of Sc_2_CF_2_-VF at positive bias is 493 μF/cm^2^ (0.40 V). It is noteworthy that the *C_Q_* of all systems with vacancies at the Fermi level is larger than that of the original system. Rui et al. investigated the *C_Q_* of doped Sc_2_CF_2_ with 13 transition metal atoms (Figure 4d) [112]. Fe-doped Sc_2_CF_2_ shows a symmetry *C_Q_*-*V* curve, with a maximum *C_Q_* of 5407.6 μF/cm^2^ at 0 V. All the 4d transition metal atoms-doped Sc_2_CF_2_ structures show asymmetric *C_Q_*-*V* curves. The maximum *C_Q_* of the Mo-doped system at negative bias is 6917.88 μF/cm^2^ at −0.2 V. For the Nb-doped system, the maximum *C_Q_* is 2599.72 μF/cm^2^ at 0.52 V under positive bias (Figure 4e). For 5d transition metal dopants, the maximum *C_Q_* is 1833.15 μF/cm^2^ (0.48 V) in the Re-doped system. Cui et al. also studied the geometry, electronic properties, and *C_Q_* of Sc_2_CF_2_ with intrinsic defects [113]. They found that the *C_Q_* fluctuations are more pronounced for systems with defects. Among them, the maximum *C_Q_* of Sc_2_CF_2_-dF→C and Sc_2_CF_2_-dC→Sc monolayers are 924.69 μF/cm^2^ (−0.48 V) and 1024.03 μF/cm^2^ (0.56 V), respectively (Figure 4f). In their study, the charge (*Q*) of the Sc_2_CF_2_-dF→C monolayer was mainly stored in the negative potential (Figure 4g).

#### 3.2.3. Hf_n+1_C_n_T_x_

Most functionalized MXenes have metallic properties, while Hf_2_CO_2_ has a moderate band gap and good thermal conductivity [114,115].

**Figure 5:**
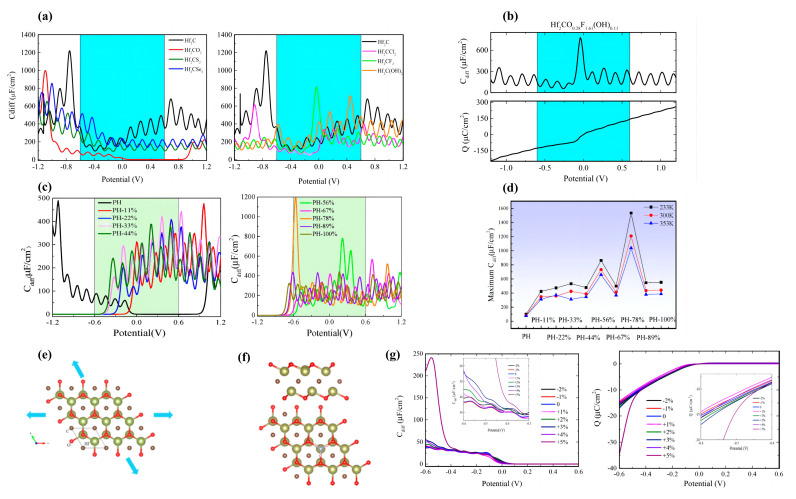
(**a**) The plot of *C_Q_* versus potential for Hf_2_CT_2_ (T = -O, -F, -S, -Cl, -OH, -Se). (**b**) The plots of *C_Q_* and surface charge versus potential for the mixed terminal of -O, -F, and -OH groups in Hf_2_C. (**c**) *C_Q_* versus potential plot of Hf_2_CO_2_ with different N-doping concentrations. (**d**) The maximum *C_Q_* of Hf_2_CO_2_ with different N-doping concentrations at 233 K, 300 K, and 353 K (**e**) Top and side views of the Hf_2_CO_2_ monolayer, where the applied biaxial strain is along the *a* and *b* directions. (**f**) Schematic diagram of the site of the NH_3_ molecule adsorption. (**g**) *C_Q_* and surface charge versus potential plots for NH_3_-adsorbed Hf_2_CO_2_ under different strain magnitudes.

Liu et al. studied the *C_Q_* of Hf_2_CT_2_ (T = -O, -F, -S, -Cl, -OH, -Se) [116]. Their results show that the maximum *C_Q_* value of Hf_2_C is 549 μF/cm^2^ (0.56 V). At positive bias, the *C_Q_* of the other functionalized systems (T = -O, -S, -Se) are smaller than the original system. The *C_Q_* of Hf_2_CO_2_ is almost zero at positive bias. The maximum *C_Q_* of Hf_2_CSe_2_ at negative bias is 564 μF/cm^2^ (−0.56 V). The maximum *C_Q_* of Hf_2_C(OH)_2_ is 804 μF/cm^2^ at 0.44 V (Figure 5a). In experiments, mixed terminations are often randomly attached to the surface of MXenes during etching; thus, there are various configurations of surface coverage and mixed terminations. The surfaces of MXenes are often bound by mixed terminations, mainly -O, -F, and -OH [117]. Therefore, Liu et al. considered MXene groups with a mixed termination of -O, -F, and -OH [116]. The results show the symmetrical characteristics of *C_Q_*-*V* curves for all three groups, with the highest *C_Q_* at zero potential of 778.82, 552.17, and 177.97 μF/cm^2^, respectively (Figure 5b).

Liu et al. also computationally investigated the effect of N doping concentration on the electronic properties and *C_Q_* of Hf_2_CO_2_ [118]. Based on the calculation results, the *C_Q_* of Hf_2_CO_2_ systems with doping concentrations of 11%, 22%, 33%, and 44% are relatively low at negative bias. The maximum *C_Q_* of the pristine Hf_2_CO_2_ system (PH) is 84.06 μF/cm^2^. The maximum *C_Q_* values of PH-33% and PH-100% at positive bias are 423.62 μF/cm^2^ and 441.16 μF/cm^2^, respectively. The maximum *C_Q_* of PH-78% is 1208 μF/cm^2^. Thus, it indicates that the N-doping concentration also changes the *C_Q_* of Hf_2_CO_2_ monolayer. (Figure 5c) Subsequently, Liu et al. further investigated the maximum *C_Q_* of PH and the doped systems at different temperatures [118]. They noted that the maximum *C_Q_* decreased with increasing temperature for all systems except PH-22%. Among all systems, the maximum *C_Q_* is 1535.2 μF/cm^2^ at 233 K for the PH-78% system (Figure 5d). As with their previous studies, they also considered the case of mixed terminals. Their results show that the maximum *C_Q_* is increased for all other systems after N-doping. As the doping concentration increases, the *C_Q_*-*V* curve becomes more symmetrical (in the range of ±0.6 V).

More interestingly, Cui et al. explored the *C_Q_* of Hf_2_CO_2_ monolayers under bi-axial strain [119]. Strain is a common strategy to modulate the properties of materials, which can tune the electronic structure of the material, thus affecting many physical properties of the material. For example, it has been experimentally demonstrated that the introduction of tensile strain can lead to a transition from direct to indirect bandgap in the MoS monolayer, which expands the light absorption range and reduces the complexation of photogenerated carriers. Cui et al. have demonstrated that strain can significantly modulate the electronic structure of Hf_2_CO_2_ monolayers, which has a very important impact on the material properties [119]. The results show that the maximum *C_Q_* values of strain-free Hf_2_CO_2_ are 1.57 μF/cm^2^ (0 V) and 78.99 μF/cm^2^ (−0.6 V) under positive and negative bias, respectively. Under positive bias, the maximum *C_Q_* of the Hf_2_CO_2_ monolayer increases at all strains except 3%. Under negative bias, the maximum *C_Q_* of the Hf_2_CO_2_ monolayer increases at all strains except −6%, −4%, and −2% (Figure 5e). Li et al. explored the effect of adsorption of NH_3_ on pristine Hf_2_CO_2_ and varying its biaxial stress [120]. In the range of ±0.6 V, the *C_Q_*-*V* curve of Hf_2_CO_2_ under strain is asymmetric. The *C_Q_* at negative potentials is significantly higher than that at positive potentials, and the maximum *C_Q_* of Hf_2_CO_2_ under free strain is 38.75 μF/cm^2^ at −0.57 V. The maximum *C_Q_* increases gradually with increasing tensile strain and reaches a maximum of 244.27 μF/cm^2^ at +5% strain (Figure 5f,g).

#### 3.2.4. Zr_n+1_C_n_T_x_

Zr_2_CO_2_ is an excellent functionalized MXene with many excellent properties. Due to its excellent photovoltaic properties and high hole mobility, it can be considered as a suitable photocatalyst [121,122].

**Figure 6:**
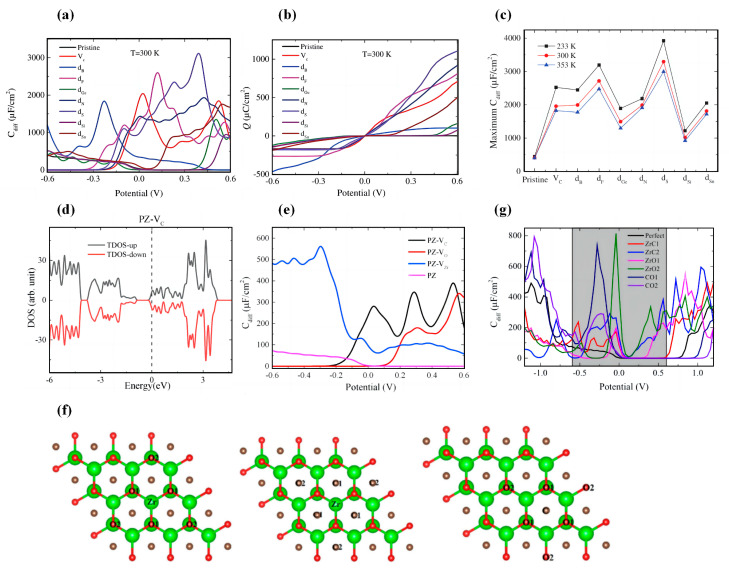
(**a**,**b**) *C_Q_* and surface charge versus potential curves for the doped or vacant Zr_2_CO_2_. (**c**) The maximum *C_Q_* of pristine Zr_2_CO_2_, Zr_2_CO_2_-V_C_, and doped Zr_2_CO_2_ at different temperatures. (**d**) DOS of a C-vacant Zr_2_CO_2_ monolayer (PZ-V_C_). (**e**) shows the *C_Q_* versus potential of PZ-V_C_, PZ-V_O_, and PZ-V_Zr_. (**f**) Schematic diagram of Zr_2_CO_2_ with different atomic exchange modifications. (**g**) *C_Q_* versus potential plot for atomic exchanged Zr_2_CO_2_.

Xu et al. investigated the electronic properties and *C_Q_* of pristine, doped, and single C vacant (VC) Zr_2_CO_2_ [123]. The doped atoms were chosen as Y = Si, Ge, Sn, N, B, S, F (Figure 6a,b). The doped atoms had a significant effect on *C_Q_* and *Q*. The maximum *C_Q_* of pristine Zr_2_CO_2_ was 407 μF/cm^2^ (−0.6 V) and 32.3 μF/cm^2^ at the Fermi level. The introduction of C vacancies increased the *C_Q_* at positive bias. The introduction of all the considered dopant atoms increased the maximum *C_Q_*, and B doping at negative bias increased the maximum *C_Q_* to 1993 μF/cm^2^. The maximum *C_Q_* of S-doped structure was 3293.7 μF/cm^2^ (0.4 V). At 0 V, a significant increase in *C_Q_* can be observed in the systems doped with VC, F, N and S atoms. Xu et al. also explored the maximum *C_Q_* of pristine Zr_2_CO_2_, Zr_2_CO_2_-VC, and doped Zr_2_CO_2_ at different temperatures [123]. Similar to the trend of *C_Q_* with temperature for N-doped Hf_2_CO_2_, they noted that the maximum *C_Q_* of each of the studied Zr_2_CO_2_ systems decreased gradually with increasing temperature. (Figure 6c)

Li et al. investigated the effect of C, O, and Zr vacancies (V_C_, V_O_, V_Zr_) on the *C_Q_* of Zr_2_CO_2_ monolayers [124]. Their study shows that the introduction of atomic vacancies increases the maximum *C_Q_* in the range of ±0.6 V. In particular, the maximum *C_Q_* of PZ-VZr at positive bias is 586 μF/cm^2^ (−0.30 V). The maximum *C_Q_* of PZ-VC MXene under positive bias is 422 μF/cm^2^ (0.53 V), while the maximum *C_Q_* of PZ-VO MXene is 359 μF/cm^2^ (0.57 V) (Figure 6d,e). Yin et al. investigated the *C_Q_* of Zr_2_CO_2_ with an atomic exchange [125]. According to the plot of *C_Q_*-*V*, they point out that, in the range of ±0.6 V, the *C_Q_* of the pristine Zr_2_CO_2_ tends to be zero in positive bias, and in negative bias, the highest *C_Q_* is 76.8 μF/cm^2^. The highest *C_Q_* is 737.1 μF/cm^2^ in negative bias for the C-O1 exchanged system and 814.6 μF/cm^2^ in negative bias for the Zr-O_2_ exchanged system. The atomic exchange in Zr_2_CO_2_ greatly improves the top *C_Q_* at negative bias in ±0.6 V. The Zr-O_2_ exchanged system has the highest *C_Q_* of 425.3 μF/cm^2^ at 0 V (Figure 6f,g).

#### 3.2.5. Nb_n+1_A_n_T_x_

The first synthesized 2D niobium carbide was the thicker Nb_4_C_3_. The Nb_2_C and Nb_3_C_2_ systems have an extremely high theoretical capacitance of Li atoms. Additionally, the surface termination has a considerable effect on the energy storage performance [126].

**Figure 7:**
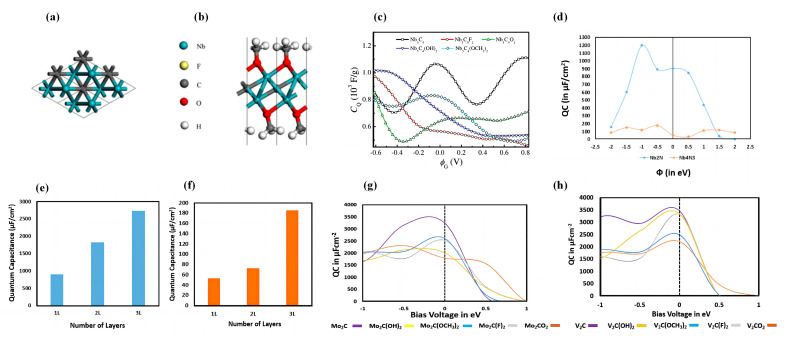
(**a**) Top view of Nb_2_C structure. (**b**) Side view of Nb_2_C(OCH_3_)_2_. (**c**) *C_Q_* versus potential curves of Nb_5_C_4_ adsorbed with different functional groups. (**d**) Variation in *C_Q_* for pristine unpolarized niobium nitride structures under bias voltage. (**e**,**f**) *C_Q_* of multilayered Nb_2_N and Nb_4_N_3_. (**g**,**h**) *C_Q_* versus potential curves for functionalized Mo_2_C and V_2_C.

Xin et al. explored the *C_Q_* properties of different thicknesses of Nb*_n_*_+1_C*_n_* (*n* = 2, 3, 4) [127]. They noted that the *C_Q_* values of all intrinsic niobium carbides were higher than that of functionalized ones in the positive bias voltage range. Except for Nb_5_C_4_, the other three niobium carbides have higher *C_Q_* values at positive bias than at negative bias. The functionalized Nb*_n_*_+1_C*_n_* shows a higher *C_Q_* than the intrinsic system only at potentials below −0.4 V. In the positive bias range, functionalization causes a significant decrease in the *C_Q_* of the system. To quantitatively compare the *C_Q_*, they calculated the theoretical integrated *C_Q_* of the positive and negative electrodes from 0 to 0.83 V and from −0.62 to 0 V, respectively. Their results illustrate that the *C_Q_* of the intrinsic niobium carbide gradually decreases with the increasing number of layers in the positive potential region. For different thicknesses of functionalized Nb*_n_*_+1_C*_n_* MXenes, the *C_Q_* is smaller than the intrinsic state. The *C_Q_* of intrinsic Nb_2_C is up to 1828.4 F/g at the positive electrode and 1091.1 F/g at the negative electrode (Figure 7a–c).

Transition metal nitrides, such as vanadium nitride, titanium nitride, and tungsten nitride, have been studied as electrode materials for EDLCs. It has been demonstrated that cobalt doping can increase the capacitance of niobium nitride. Bharti et al. calculated the *C_Q_* of Nb_2_N and Nb_4_N_3_ and investigated the effect of Co-doping on their *C_Q_* [128]. Their calculations show that the *C_Q_* value of Nb_2_N is remarkably high (1196.28 μF/cm^2^, −1 V) and the *C_Q_* of Nb_4_N_3_ is much lower than that of Nb_2_N. When they increased the number of layers of Nb_2_N and Nb_4_N_3_, they found that the *C_Q_* kept increasing with the more layer numbers. The *C_Q_* of both Nb_4_N_3_ (Nb_4_N_3_-2Co) and Nb2N (Nb_2_N-2Co) increase after Co-doping at the Fermi level, with the *C_Q_* of Nb_2_N-2Co reaching 1052.2 μF/cm^2^ (Figure 7d–f).

#### 3.2.6. Mo_2_C and V_2_C

Two-dimensional transition metal carbides (TMCs) have high melting points and good electrical conductivity and chemical stability [129,130,131]. With its excellent electrochemical properties, Mo_2_C has been experimentally prepared as an electrode material for capacitors. In a experiment prepared by Lu et al., the capacitor with Mo_2_C as the electrode material had a high specific capacitance and excellent cycling stability, and its performance was significantly better than most carbide-based asymmetric supercapacitors [132]. As early as 2015, it was shown that V_2_CT*_x_* could be used as the positive electrode of sodium ion capacitors [133]. The results of Ai et al.’s study show that the specific capacitance of V_2_C in 1 M Na_2_SO_4_ is high (223.5 F/g) and the cycling stability is good (capacitance retention could be maintained at 94.7% after 5000 cycles) when the current density is 100 mA/g [134].

Bharti et al. discussed the *C_Q_* of Mo_2_C and V_2_C [135]. They highlighted that the *C_Q_* of intrinsic Mo_2_C and V_2_C reaches 3243.99 μF/cm^2^ and 3465.51 μF/cm^2^ at the Fermi level, respectively. In the positive potential range, the *C_Q_* decreases rapidly and drops to 0. Similar to Nb_2_N and Nb_4_N_3_, the *C_Q_* of Mo_2_C and V_2_C enhances with the increasing number of layers. The *C_Q_* values of both Mo_2_C and V_2_C at the Fermi energy decreased after O-functionalization. Similar to the pristine system, the O-functionalized V_2_C and Mo_2_C had higher *C_Q_* at negative bias (Figure 7g,h).

### 3.3. Transition Metal Dichalcogenides (TMDs)

Transition metal-based materials are considered to have higher energy density than other materials [136,137,138,139]. Among them, transition metal dichalcogenides (TMDs) are a class of graphene-like structures that have been commonly used in terms of electrode materials for supercapacitors in recent years. Although TMDs usually store energy in the form of intercalation with alkali metals, they exhibit quantum effects that are reflected in their capacitive behavior.

MoS_2_ is a typical representative of TMDs and exists in three main phases (2H, 3R, and 1T) with unique capacitive properties. Graphene-like MoS_2_ materials have special structures, fast ionic conductivity, and high specific capacitance [140,141]. In addition, the electron correlation between Mo layers in the sandwich structure facilitates the carrier transport [142,143,144]. It is an excellent electrode material for supercapacitors and has attracted more and more attention.

**Figure 8:**
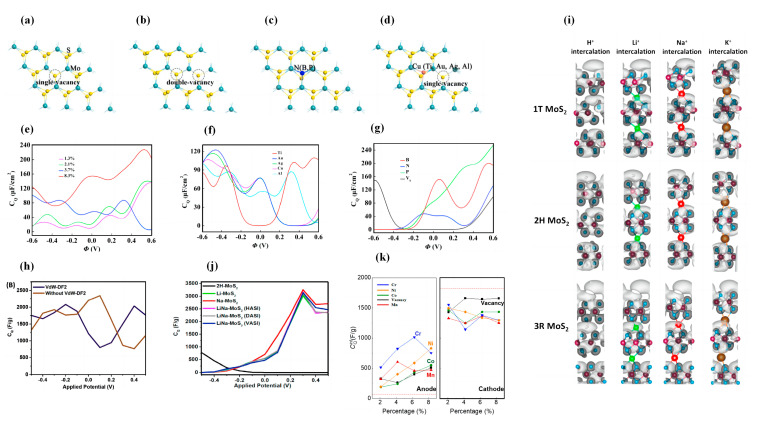
Structures of (**a**) single-vacant (VS) MoS_2_ monolayer; (**b**) double-vacant (V_2_S) MoS_2_ monolayer; (**c**) B, N, P, Ti, Au, Ag, Cu, and Al-doped MoS_2_ monolayer; (**d**) Ti, Au, Ag, Cu, and Al-doped VS-MoS_2_ monolayer. (**e**) The plot of *C_Q_* versus potential for VS-MoS_2_ monolayers with Al substituted S atoms at different doping concentrations. (**f**) *C_Q_* versus potential plot for VS-MoS_2_ monolayers with Ti-, Au-, Ag-, Cu-, and Al-substituted S atoms. (**g**) The plot of *C_Q_* versus potential for pristine MoS_2_ monolayers with B-, N-, and P-substituted S atoms. (**h**) Plots of *C_Q_* versus potential with and without vdW for three-layered 1T-phase MoS_2_. (**i**) Plots of differential charge density for 1T, 2H, and 3R-phases MoS_2_. (**j**) *C_Q_* versus potential curves for Li^+^ and Na^+^ co-doped 2H phase MoS_2_. (**k**) *C_int_* of *h*-FeS for anode-like and cathode-like supercapacitors at different doping concentrations.

MoS_2_ was the subject of a comprehensive study by Xu et al. [145]. They first investigated the relationship between the *C_Q_* and the potential of MoS_2_ containing different dopants (where Ti, Au, Ag, Cu, and Al replace Mo atoms), single-vacancy V_Mo_. Their results show that the *C_Q_* of pristine MoS_2_ is almost zero in the region near 0 V and increases at a higher voltage. For the *C_Q_* of Al-doped MoS_2_ and single-vacancy V_Mo_, the local *C_Q_* maxima near 0 V are 157.7 μF/cm^2^ and 156.5 μF/cm^2^, respectively. Subsequently, they observed the effect of Al doping on the *C_Q_* in both pristine and single-vacant (VS) MoS_2_ monolayers with different Al concentrations of 1.3%, 2.1%, 3.7%, and 8.3%. The *C_Q_* value increases from 44.76 μF/cm^2^ (0.17 V) to 227.85 μF/cm^2^ (0.53 V) with the increase in doping concentration (Figure 8a–e). Secondly, they investigated the doped VS-MoS_2_, where Ti, Au, Ag, Cu, and Al replace the S atoms. The *C_Q_* increased in all systems except when doped with Ti, which is similar to that of S substitution in pristine MoS_2_ (Figure 8f). Finally, they calculated the local *C_Q_* maxima of 200.89 μF/cm^2^, 132.77 μF/cm^2^, and 254.29 μF/cm^2^ in B-, N-, and P-substituted S atoms in the B-, N-, and P-doped MoS_2_ monolayer (doping concentration kept at 3.7%), respectively. At the Fermi level, the B-doped system had higher *C_Q_* and a clear advantage in terms of positive potential (Figure 8g). Therefore, they continued to investigate the effect of B-doping concentration on *C_Q_*. The *C_Q_* value increased gradually with the increase of B-doping concentration.

It is worth noting that MoS_2_ should be considered as a van der Waals (vdW) 2D material. Biby et al. investigated the *C_Q_* of multilayered MoS_2_ with embedding and co-embedding in relation to vdW forces [146]. The *C_Q_* of the three-layered 1T phases was as high as 2080 F/g, and the *C_Q_* of 3R-MoS_2_ was slightly higher than that of 2H-MoS_2_ under negative bias. Subsequently, in their investigation on the effect of vdW forces on the *C_Q_*, they pointed out that the absence of vdW forces increased the strength of the density of electric states. Thus, for 1T-MoS_2_, the absence of vdW leads to a higher *C_Q_* at positive bias, and in the case of 2H and 3R-MoS_2_, the absence of vdW shifts the Fermi level, leading to a higher *C_Q_* in the negative potential window. This finding also directly emphasizes the importance of vdW forces in the accurate calculation of 2D material properties (Figure 8h,i). Finally, they investigated the intercalation of cations (Li^+^, Na^+^, K^+^ and H^+^) in the three phases of MoS_2_ and mixed Li^+^ and Na^+^ intercalation. They conclude that the *C_Q_* of the 1T phase is increased near the Fermi level with Na^+^ intercalation. Additionally, the 2H and 3R phases have a larger improvement, mainly in the positive bias voltage. There are three different intercalation modes in the case of mixed doping, and the *C_Q_* values of the co-intercalated system are higher than 2H Li-MoS_2_ and close to 2H Na-MoS_2_. The maximum *C_Q_* of LiNa-MoS_2_ (HASI) can reach 3163 F/g (Figure 8j) [146].

Irham et al. showed that the introduction of defects in h-FeS increased the *C_Q_* at positive bias up to 2280 F/g, but the *C_Q_* at negative bias decreased [147]. Cr-doped FeS has a maximum *C_Q_* of 3076 F/g (0.6 V) at positive bias. *P*-type dopants (Co or Ni) do not significantly increase the *C_Q_* in the positive voltage range. In their study of the integrated *C_Q_*, they found that the *C_int_* also changes nonlinearly with the doping concentration at positive bias, where the *C_int_* can reach 1013 F/g with the Cr-doping concentration of 6.24%. They attribute the emergence of these nonlinear changes to the appearance of electronic off-domain states that hinder the increase of *C_int_* (Figure 8k).

## 4. Conclusions

In this paper, we reviewed studies investigating the *C_Q_* of 2D electrode materials through using theoretical calculations. In general, there are two solutions to enhance the performance of electrode materials for supercapacitors. One is to develop new electrode materials with higher performance, and the other is to modify the already found electrode materials, mainly by means of doping, adsorption, defects, atom exchange, etc. As far as the available studies are concerned, for graphene-like main group elements and compounds, all of the modification measures mentioned above can improve the *C_Q_* of the electrode material. In contrast, in MXene materials, not all modification measures are able to improve the material performance. For example, the *C_Q_* of functionalized Ti_3_C_2_ is not higher than that of the pristine structure. However, the introduction of functional groups during the preparation process is inevitable. Thus, it is necessary to investigate the *C_Q_* of the functionalized MXenes to select the preparation precursors with the least impact on energy storage performance. There are not many studies on *C_Q_* in transition metal-based supercapacitor electrode materials. Most TM-based materials are considered as pseudocapacitance supercapacitor materials. However, both pseudocapacitance and electric double-layer processes exist in such supercapacitors. So calculating the *C_Q_* of TM-based electrode materials can lead to a more accurate prediction of the theoretical capacitance and provide more possibilities for the development of supercapacitors.

Nowadays, theoretical calculation plays an important role in scientific research. On the one hand, theoretical calculations can help interpret the results of existing experimental phenomena, and on the other hand, they facilitate the prediction and development of new materials. Most of the current studies on the theoretical calculation of electrode materials for supercapacitors focus on predicting new materials. Although the high performance of electrode materials has been calculated theoretically, there are still great difficulties in the preparation process. In addition, the current research only pertains to the electrode part, and the performance of the whole capacitor has not been completely considered. Further research should pay more attention to the feasibility and stability of material modification. Secondly, various variables that may cause influence should be fully considered so that the theoretical prediction is closer to the real situation. And thirdly, the amount of attention given to the overall performance and process of supercapacitors should be improved. With the advancement of science and technology, theoretical calculations have become more accurate and fast. This also provides us with a more thorough explanation of supercapacitor performance and greater feasibility for designing functional materials with various properties. We expect that theoretical calculations will provide a greater contribution to the development of supercapacitors.

## Data Availability

No new data were created or analyzed in this study. Data sharing is not applicable to this article.
